# Disparities in health condition diagnoses among aging transgender and cisgender medicare beneficiaries, 2008-2017

**DOI:** 10.3389/fendo.2023.1102348

**Published:** 2023-03-13

**Authors:** Jaclyn M. W. Hughto, Hiren Varma, Gray Babbs, Kim Yee, Ash Alpert, Landon Hughes, Jacqueline Ellison, Jae Downing, Theresa I. Shireman

**Affiliations:** ^1^ Center for Health Promotion and Health Equity, Brown University School of Public Health, Providence, RI, United States; ^2^ Departments of Behavioral and Social Sciences and Epidemiology, Brown University School of Public Health, Providence, RI, United States; ^3^ The Fenway Institute, Fenway Health, Boston, MA, United States; ^4^ Center for Gerontology & Healthcare Research, Brown University School of Public Health, Providence, RI, United States; ^5^ Department of Health Services Policy & Practice, Brown University School of Public Health, Providence, RI, United States; ^6^ Oregon Health & Science University - Portland State University School of Public Health, Portland, OR, United States; ^7^ Department of Public Health Sciences, University of Rochester Medical Center, Rochester, NY, United States; ^8^ Department of Health Behavior and Health Education, University of Michigan School of Public Health, Ann Arbor, MI, United States; ^9^ Institute for Social Research, University of Michigan, Ann Arbor, MI, United States; ^10^ Department of Health Policy and Management, University of Pittsburgh School of Public Health, Pittsburgh, PA, United States; ^11^ Center for Innovative Research on Gender Health Equity (CONVERGE), University of Pittsburgh Department of Medicine, Pittsburgh, PA, United States

**Keywords:** transgender, Medicare, aging, older adults, insurance claims, chronic conditions, health disparities, diagnoses

## Abstract

**Introduction:**

The objective of this research is to provide national estimates of the prevalence of health condition diagnoses among age-entitled transgender and cisgender Medicare beneficiaries. Quantification of the health burden across sex assigned at birth and gender can inform prevention, research, and allocation of funding for modifiable risk factors.

**Methods:**

Using 2009–2017 Medicare fee-for-service data, we implemented an algorithm that leverages diagnosis, procedure, and pharmacy claims to identify age-entitled transgender Medicare beneficiaries and stratify the sample by inferred gender: trans feminine and nonbinary (TFN), trans masculine and nonbinary (TMN), and unclassified. We selected a 5% random sample of cisgender individuals for comparison. We descriptively analyzed (means and frequencies) demographic characteristics (age, race/ethnicity, US census region, months of enrollment) and used chi-square and t-tests to determine between- (transgender vs. cisgender) and within-group gender differences (e.g., TMN, TFN, unclassified) difference in demographics (p<0.05). We then used logistic regression to estimate and examine within- and between-group gender differences in the predicted probability of 25 health conditions, controlling for age, race/ethnicity, enrollment length, and census region.

**Results:**

The analytic sample included 9,975 transgender (TFN n=4,198; TMN n=2,762; unclassified n=3,015) and 2,961,636 cisgender (male n=1,294,690, female n=1,666,946) beneficiaries. The majority of the transgender and cisgender samples were between the ages of 65 and 69 and White, non-Hispanic. The largest proportion of transgender and cisgender beneficiaries were from the South. On average, transgender individuals had more months of enrollment than cisgender individuals. In adjusted models, aging TFN or TMN Medicare beneficiaries had the highest probability of each of the 25 health diagnoses studied relative to cisgender males or females. TFN beneficiaries had the highest burden of health diagnoses relative to all other groups.

**Discussion:**

These findings document disparities in key health condition diagnoses among transgender Medicare beneficiaries relative to cisgender individuals. Future application of these methods will enable the study of rare and anatomy-specific conditions among hard-to-reach aging transgender populations and inform interventions and policies to address documented disparities.

## Introduction

Transgender people in the United States (US) experience significant health disparities throughout their life course relative to cisgender (non-transgender) people (1–6). These disparities stem from multilevel sources of stigma that serve as sources of chronic stress as well as barriers to accessing essential resources such as healthcare, employment, and income (7–9). While extensive community-based research drawn from convenience samples (10, 11), and research using state or national data has assessed the health of transgender youth and adults overall (12–14) and relative to cisgender people (15–18), a dearth of national studies have compared the health of an exclusively aging (i.e., age 65 or older) sample of transgender and cisgender adults.

The risk of being diagnosed with a chronic health condition, such as cancer, HIV, depression, osteoporosis, and dementia, increases as one ages (19, 20). Aging transgender adults are expected to be at an even higher risk of developing physical and mental health conditions than their cisgender peers due to stigma-related stress experienced throughout their lives and barriers to accessing quality healthcare (9, 21–25). Although researchers are increasingly studying the health of aging transgender adults (26–28), and some national studies have explored disparities among predominantly aging populations relative to cisgender groups (29–32), no national research, to our knowledge, has explored within- (e.g., trans feminine people vs. trans masculine people) and between- (e.g., transgender vs. cisgender) group gender differences among aging transgender adults and a general population of cisgender adults. Without comparative data on the health of aging transgender and cisgender subpopulations, it is difficult to know which subgroups are in greatest need of public health and policy interventions.

Recent methodological advances have enabled the use of claims databases to study the health of transgender populations. Blosnich et al. (2013) innovatively used transgender-specific International Classification of Diseases, Ninth Edition (ICD-9) diagnosis codes (e.g., Gender Identity Disorder (GID)) to identify transgender veterans using national Veteran Health Administration data (14). Proctor and colleagues later followed by applying transgender-related ICD-9 diagnosis codes to identify age- and disability-entitled transgender Medicare beneficiaries (33). Using the same approach as Proctor et al., Dragon and colleagues found that, compared to cisgender Medicare recipients receiving care in 2015, a higher proportion of transgender beneficiaries receiving care during the sample year had been diagnosed with several major health conditions, including asthma, autism spectrum disorder, chronic obstructive pulmonary disease, depression, hepatitis, HIV, schizophrenia, and substance use disorders (30). Building on the work of Proctor and Dragon, Progovac and colleagues examined disparities between transgender and cisgender Medicare beneficiaries receiving care between 2009 and 2014 (31). They found that older and disabled transgender Medicare beneficiaries had more diagnoses for chronic health conditions than their cisgender counterparts (31). Notably, however, none of these studies examined health diagnoses disparities by gender subgroup (trans feminine, trans masculine, cisgender male, cisgender female), despite prior survey-based research showing substantial within and between gender group variations in mental and physical health conditions (2, 6, 10, 11, 34, 35).

Our team has advanced algorithms identifying transgender beneficiaries in claims data and inferring their gender (12, 13, 36). Using commercial insurance data from 2001-2019, we adapted a method developed by Jasuja and colleagues (13) that used diagnosis, procedure, and pharmacy claim codes to identify individuals with one or more transgender-related diagnoses or Endocrine Disorder Not Otherwise Specified [Endocrine NOS] in conjunction with prescriptions for gender-affirming hormones (e.g., estrogen, testosterone) or gender-affirming procedures (e.g., phalloplasty, vaginoplasty) to identify 38,598 transgender adults. We furthered the algorithm using an approach developed by Yee, Lind, & Downing (36) for Oregon Medicaid recipients to improve our ability to categorize transgender samples by inferred gender.^1^ Specifically, we used a hierarchical approach to examine beneficiaries’ history of gender-affirming and reproductive anatomy-specific^2^ procedures (e.g., prostate-related procedures, hysterectomy), diagnoses, and pharmacy claims for gender-affirming hormones to categorize the sample based on inferred gender identity:^3^ trans masculine/nonbinary (TMN) or trans feminine/nonbinary (TFN) (12).^4^ By including reproductive anatomy-specific procedures and diagnoses in combination with gender-affirming care, we were able to infer the gender of 76% of the sample (50% TMN; 26% TFN), which represented a notable improvement from prior approaches (13).

When we applied our modified algorithm to study the health of younger, commercially-insured transgender individuals (12), we found that relative to TMN people, TFN people had significantly higher predicted probabilities of most health condition diagnoses, including HIV, atherosclerotic cardiovascular disorder, myocardial infarction, alcohol use disorder, and substance use disorder (4). In contrast, TMN individuals had significantly higher predicted probabilities of diagnosed post-traumatic stress disorder and depression than TFN people. While our prior research provides insights into within-group gender-related disparities in health diagnoses among commercially-insured adults aged 18 and over, it is unknown whether these patterns are similar among aging transgender adults aged 65 and older. To more fully understand the health of aging transgender adults, research is needed to explore within- and between-group gender differences in health diagnoses among aging transgender adults, overall and relative to their cisgender counterparts. As the largest insurer of U.S adults aged 65 and older (37), Medicare is the ideal data source to utilize to document within- and between-group disparities in health diagnoses for aging transgender and cisgender adults.

Building on prior work (13, 31, 36), in the present study, we sought to apply our claims-based method (12) to identify transgender and cisgender samples in Medicare data, stratify the samples by gender, and explore within- and between-group gender differences in health diagnoses among aging transgender and cisgender Medicare beneficiaries. Findings from this national study can help identify health diagnosis disparities among aging Medicare beneficiaries and the subgroups in greatest need of tailored interventions to prevent and treat adverse health outcomes.

## Materials and methods

### Study design/data source

We conducted a retrospective cross-sectional analysis to identify transgender adults, stratify them into inferred gender subgroups, and compare these groups to cisgender people. Fee-for-service Medicare data were accessed through the Virtual Data Resource Center (VRDC) maintained by the Centers for Medicare & Medicaid Services (CMS) through a data use agreement (DUA 52772). We queried the Medicare Master Beneficiary Summary File and final paid claims for inpatient, physician, and other suppliers, and prescription services from 2008 to 2017.

### Identifying transgender individuals

To identify transgender individuals, we adapted our algorithm (12) developed with commercial insurance claims for Medicare. These methods and the corresponding codes used to identify the transgender sample are described in detail elsewhere (12, 13). Briefly, we included any person with a transgender-related diagnosis (e.g., GID); transgender-conclusive procedures (e.g., “operations for sex transformation, not elsewhere classified”); a diagnosis of endocrine NOS in conjunction with a transgender-suggestive procedure or gender-affirming hormone prescription (**Supplementary Figure A**).

### Stratifying the transgender sample by inferred gender

We subsequently applied a previously-developed stepwise approach (12) to categorize the inferred gender of the transgender sample (**Supplementary Figure B**). As described in detail elsewhere (12), briefly, we first classified the inferred gender of transgender individuals based on the presence of claims for gender-affirming genital surgeries (e.g., “vaginal construction,” “construction of penis”). Then, within the remaining sample, we categorized the sample by gender if they had certain types of highly specific and highly sensitive reproductive anatomy-specific care and diagnoses (e.g., hysterectomy, pregnancy, prostate cyst, prostate screening). Next, we categorized individuals according to their receipt of gender-affirming hormones or procedures. Finally, using the remaining sample, individuals who had other reproductive anatomy-related diagnoses or procedures (e.g., vulvectomy for TMN or testicular hyperfunction for TFN) were categorized by inferred gender. Individuals who had not yet been assigned a gender category or those with conflicting codes at the final step remained unclassified. The unclassified group was comprised of people with a transgender-related diagnosis code (e.g., GID, transsexualism) and no gender-affirming hormones or procedures or reproductive-anatomy-related care or who had conflicting codes.

### Eligibility criteria for cisgender comparison cohort

After identifying the transgender cohort, we selected a random 5% sample from the remaining Medicare beneficiaries, whom we refer to here and going forth as cisgender. The sex of the beneficiaries classified as cisgender was taken from the Master Beneficiary Summary File. Because identification of the transgender cohort relied on engagement in care, we limited the cisgender cohort to beneficiaries who had at least one Part A, B, or D claim between 2008 and 2017. We excluded cisgender beneficiaries with missing data on sex and/or date of birth (about 5% of the sample).

### Measures

*Sociodemographics*. Age was categorized as 65-<70; 70-74; 75-79, 80-84, 85+. Race and ethnicity were categorized as Asian (non-Hispanic), Black (non-Hispanic), Hispanic, White (non-Hispanic), another race/ethnicity (non-Hispanic), or unknown. US Census regions included Northeast, Midwest, South, West, and Unknown. Since all Medicare beneficiaries receive fee-for-service (FFS) coverage and some may elect to pay for supplemental Part D prescription coverage, we created separate continuous months of insurance coverage variables for individuals with FFS coverage only and those with FFS plus Part D coverage.

*Health Condition Diagnoses.* We used the CMS Chronic Condition Warehouse to identify diagnoses for 25 health conditions (38). We grouped the health conditions diagnoses as follows: cancer (breast, colorectal, endometrial, lung, prostate); heart, lung, & kidney conditions (asthma, chronic kidney disease, chronic obstructive pulmonary disease [COPD], cardiac arrhythmia, congestive heart failure, coronary artery disease, hyperlipemia, hypertension, stroke); infectious diseases (hepatitis, HIV/AIDS); other health conditions (arthritis, diabetes, osteoporosis); mental or cognitive illness (dementia, depression, schizophrenia); and substance use disorders (alcohol, drug, tobacco use).

### Data analysis

Since Medicare data include individuals who qualify for coverage based on age (i.e., 65 or older) and disability, and the current analysis focuses on the health of aging individuals, we restricted the transgender and cisgender samples to those eligible for Medicare based on age at enrollment. We then descriptively analyzed demographic characteristics and used χ2 tests and t-tests to assess between-group (transgender vs. cisgender) and within-group differences (e.g., TMN vs. TFN) in sociodemographics (p<0.05). Next, we estimated the crude prevalence of each condition stratified by all gender subgroups. Since the distribution of sociodemographic characteristics varied by gender subgroup (TFN vs. TMN, cisgender male vs. cisgender female), we fit logistic regression models predicting the log odds of each condition while controlling for age at enrollment, race/ethnicity, months of enrollment, and Census region. To facilitate within- and between-group comparisons, we obtained the predicted probability of each condition for each of the 4 gender subgroups that could be classified (TFN people, TMN people, cisgender males, cisgender females), holding covariates at their means. Means differences in the predicted probabilities were also assessed (p<0.05). All analyses were conducted using SAS 9.4 (SAS Institute, Cary, NC).

### Reporting and discussion of results

In reporting and discussing the results for the transgender and cisgender subgroups, we describe within- and between-group gender differences in the crude prevalence and means of the demographic characteristics and the predicted probabilities of the health diagnoses. For demographics, we report and discuss differences between transgender and cisgender people overall, as well as within-group differences among transgender (TFN vs. TMN) and cisgender (male vs. female) subgroups. For the predicted probabilities, we report all diagnoses and discuss the diagnoses with the widest within- and between-group disparities.

For differences in the predicted probabilities, we first discuss between-group differences in individuals presumed to have been assigned the same sex at birth (i.e., cisgender males and TFN people; and cisgender females and TMN people). Although referring to a transgender person by their assigned birth sex is not affirming or appropriate, these comparisons were made because, on a population level, cisgender and transgender people assigned the same sex at birth are typically born with similar reproductive anatomy and endogenous hormones that could impact their risk of developing specific health conditions. People assigned the same sex at birth may also be more likely to experience similar social or developmental influences in childhood and adolescence that could influence their tendency to engage in behaviors that might increase or decrease their risk of developing specific conditions.

We also discuss between-group differences between TFN people and cisgender females and between TMN people and cisgender males. We make these comparisons as the use of gender-affirming hormones and procedures may result in TFN people having hormone exposure levels and anatomies that are more closely aligned with cisgender females than cisgender males, and TMN individuals may come to have hormone exposure levels and anatomies that are more similar to cisgender males than cisgender females.

Finally, making these comparisons in the results and discussion enables us to engage with the breadth of medical, public health, psychological, sociological, and other literature documenting the prevalence of and mechanisms underlying health diagnosis disparities between individuals who were assigned the same birth sex or who share similar gender identities or expressions. Specific limitations of this approach are denoted in the discussion.

## Results

### Demographic characteristics

**Table 1** summarizes the demographic characteristics of the sample. We identified 9,975 transgender FFS beneficiaries who qualified for Medicare based on age. Overall, 4,198 (41.1%) were categorized as TFN, 2,762 (27.7%) as TMN, and the gender could not be inferred and classified for 3,015 transgender beneficiaries (30.2% unclassified). Of the 2,961,636 cisgender individuals included in the comparison sample, 1,294,690 (43.7%) were male, and 1,666,946 (56.3%) were female.

**Table 1:** Demographics at enrollment among age-entitled beneficiaries stratified by inferred gender in a national Medicare population, 2008-2017.

	TOTAL					TRANSGENDER							CISGENDER				
	N=2,971,611					N=9,975							N=2,961,636				
	Transgender N=9,975		Cisgender N=2,961,636			TFN N=4,198		TMN N=2,762		Unclassified N=3,015			Male N=1,294,690		Female N=1,666,946		
Age at Enrollment (y)	N	%	N	%	P-Value	N	%	N	%	N	%	P-value	N	%	N	$	P-Value
65-<70	6,003	60.2	1,604,745	54.2	<0.0001	2,643	63.0	1,583	57.3	1,777	58.9	<0.0001	730,794	56.4	873,951	52.4	<0.0001
70-74	1,565	15.7	464,378	15.7		684	16.3	502	18.2	379	12.6		215,803	16.7	248,575	14.9	
75-79	1,132	11.3	362,645	12.2		481	11.5	333	12.1	318	10.5		157,583	12.2	205,062	12.3	
80-84	791	7.9	277,783	9.4		276	6.6	233	8.4	282	9.4		109,575	8.5	168,208	10.1	
>= 85	484	4.9	252,085	8.5		114	2.7	111	4.0	259	8.6		80,935	6.3	171,150	10.3	
Race/ethnicity																	
Asian	237	2.4	92,031	3.1	<0.0001	95	2.3	63	2.3	79	2.6	0.001	40,329	3.1	51,702	3.1	<0.0001
Black	655	6.6	239,491	8.1		234	5.6	197	7.1	224	7.4		95,361	7.4	144,130	8.6	
Hispanic	537	5.4	222,517	7.5		231	5.5	136	4.9	170	5.6		96,718	7.5	125,799	7.5	
White	8,303	83.2	2,340,841	79.0		3,524	83.9	2,322	84.1	2,457	81.5		1,026,070	79.3	1,314,771	78.9	
Another race/ ethnicity	105	1.1	32,060	1.1		43	1.0	21	0.8	41	1.4		14,364	1.1	17,696	1.1	
Unknown	138	1.4	34,696	1.2		71	1.7	23	0.8	44	1.5		21,848	1.7	12,848	0.8	
Census Region																	
Northeast	2,264	22.7	557,113	18.8	<0.0001	947	22.6	628	22.7	689	22.9	<0.0001	237,772	18.4	319,341	19.2	<0.0001
Midwest	1,962	19.7	669,506	22.6		788	18.8	521	18.9	653	21.7		292,635	22.6	376,871	22.6	
South	3,252	32.6	1,071,084	36.2		1,388	33.1	997	36.1	867	28.8		467,104	36.1	603,980	36.2	
West	2,468	24.7	628,446	21.2		1,060	25.3	611	22.1	797	26.4		281,716	21.8	346,730	20.8	
Unknown	---	---	35,487	1.2		---	---	---	---	---	---		15,463	1.2	20,024	1.2	
Months of Coverage	Mean	SD	Mean	SD	P-Value	Mean	SD	Mean	SD	Mean	SD	P-value	Mean	SD	Mean	SD	P-Value
Mean months of FFS Coverage	76.49	41.65	50.74	44.94	<0.0001	79.99	39.47	88.32	37.08	60.76	43.79	<0.0001	49.34	44.03	51.82	45.61	<0.0001
Mean months of FFS + Part D Coverage	50.04	41.64	32.47	38.06	<0.0001	50.36	40.19	59.53	42.28	40.92	41.06	<0.001	29.16	35.78	35.03	39.54	<0.0001

This Medicare sample was restricted to those age 65 and older who qualified for Medicare based on age. TFN, Trans Feminine and Nonbinary; TMN, Trans Masculine and Nonbinary. FFS, Fee for service. For categorical variables, the p-value is based on chi-square tests (X^2^). For continuous variables, the p-value is based on t-tests. For the transgender sample, chi-square and t-test compared differences between TFN and TMN people only. Per Center for Medicare & Medicaid Services (CMS) guidelines, the number of TFN, TMN, and unclassified transgender individuals from an unknown census region are not reported as the cell sizes for one or more groups are less than 11.

There were significant between-group gender differences in the demographics of the samples. Overall, a higher proportion of transgender beneficiaries enrolled in Medicare at a younger age, were non-Hispanic White, and lived in the Northeast or West at enrollment compared to cisgender beneficiaries. Transgender individuals also had a significantly longer mean period of continuous enrollment relative to cisgender individuals (p<.0001).

There were also significant within-group gender differences. Regarding demographic differences between TFN and TMN people, although statistically significant, there were relatively small differences between these groups with regard to race/ethnicity and region. Notably, among the transgender sample, a larger proportion of TFN people enrolled before age 70, whereas a larger proportion of the unclassified group enrolled at age 85 or older; also, on average, TMN had significantly more months of continuous enrollment than other transgender groups (p<.0001). With regard to with-in-group differences for the cisgender sample, a higher proportion of cisgender males than females enrolled in Medicare before age 70, yet, on average, cisgender females had more continuous months of enrollment than cisgender males. Further, despite statistically significant differences in the distribution of race-ethnicity and geographic region, percent point differences were relatively small between cisgender males and females.

### Health condition diagnoses

**Figure 1:**
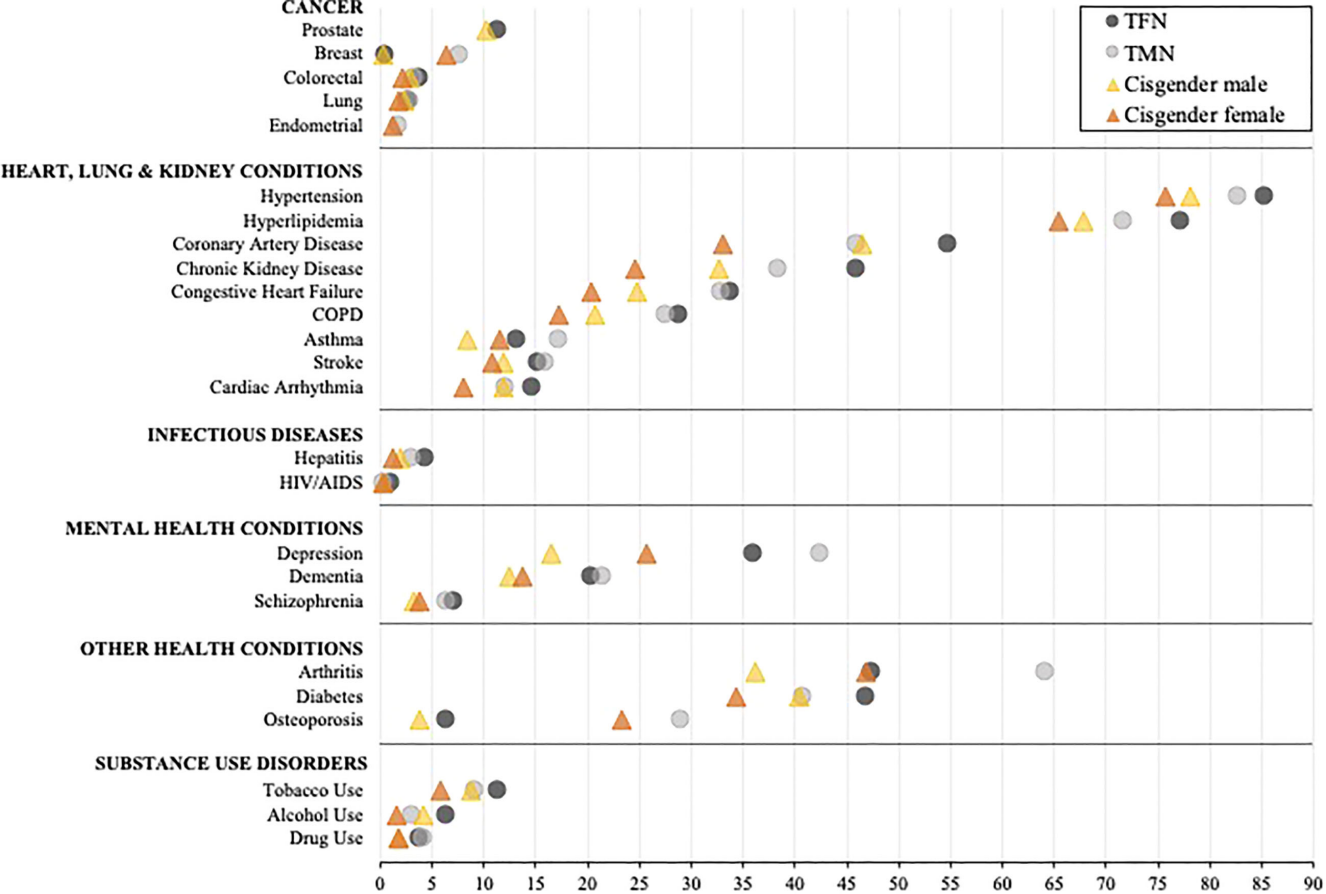
Comparison of the adjusted predicted probabilities of health condition diagnosed stratified by inferred gender among age-entitled transgender and cisgender Medicare beneficiaries, 2008-2017. TFN, Trans Feminine and Nonbinary; TMN, Trans Masculine and Nonbinary. COPD, Chronic obstructive pulmonary disease. The symbols represent the adjusted predicted probabilities when age, enrollment length, race/ethnicity, and census region are set to their means. The 95% confidence intervals are shown in **Table 2**. Within each diagnostic category, the conditions are represented from high to low based on the probability of each diagnosis for TFN individuals.

The unadjusted prevalence for each diagnosis among all transgender and cisgender subgroups is presented in **Supplementary Table 1**. **Table 2** and **Figure 1** present results from the adjusted models for TMN and TFN people and cisgender males and females, and **Supplementary Table 2** presents the mean differences in the predicted probability of each diagnosis by gender subgroup. Overall, TFN or TMN people had the highest predicted probability (herein “probability”) of every diagnosis relative to cisgender males and females. Additionally, TFN individuals had the highest probability of being diagnosed with the majority of health conditions compared to TMN people, as well as cisgender males and females.

**Table 2:** Adjusted predicted probabilities of health condition diagnoses stratified by inferred gender among age-entitled transgender and cisgender Medicare beneficiaries, 2008-2017.

	TRANSGENDER				CISGENDER			
	TFN N=4,198		TMN N=2,762		Male N=1,294,690		Female N=1,666,946	
	%	95% CI	%	95% CI	%	95% CI	%	95% CI
CANCER								
Breast	0.4	0.2-0.5	7.5	6.8-8.4	0.1	0.1-0.1	6.2	6.1-6.3
Colorectal	3.6	3.1-4.1	3.1	2.6-3.6	2.7	2.6-2.8	2.1	2.0-2.1
Endometrial	—	—	1.7	1.4-2.1	—	—	1.1	1.1-1.2
Lung	2.5	2.1-2.9	2.7	2.3-3.3	2.3	2.2-2.4	1.7	1.6-1.7
Prostate	11.2	10.4-12.1	—	—	10.1	9.9-10.3	—	—
HEART, LUNG, & KIDNEY CONDITIONS								
Asthma	13.1	12.1-14.0	17.2	16.0-18.5	8.2	8.1-8.3	11.5	11.4-11.7
Cardiac Arrhythmia	14.5	13.6-15.5	12.0	11.1-13.1	11.7	11.6-11.9	8.0	7.9-8.1
Chronic Kidney Disease	45.9	44.3-47.6	38.3	36.4-40.3	32.5	32.3-32.8	24.4	24.3-24.6
Congestive Heart Failure	33.7	32.2-35.3	32.7	30.9-34.6	24.6	24.4-24.8	20.3	20.1-20.5
COPD	28.7	27.3-30.1	27.5	25.9-29.2	20.6	20.4-20.7	17.2	17.0-17.3
Coronary Artery Disease	54.6	52.9-56.4	45.9	43.8-48.0	46.4	46.1-46.6	32.9	32.7-33.1
Hyperlipemia	77.1	75.4-78.6	71.6	69.3-73.8	67.7	67.3-67.8	65.4	65.2-65.6
Hypertension	85.2	84.0-86.3	82.6	80.8-84.3	78.1	77.9-78.2	75.7	75.5-75.9
Stroke	15.1	14.1-16.2	15.8	14.6-17.1	11.8	11.6-11.9	10.7	10.6-10.8
INFECTIOUS DISEASES								
Hepatitis	4.3	3.7-5.1	3.0	2.4-3.7	1.8	1.7-1.9	1.2	1.2-1.3
HIV/AIDS	0.9	0.6-1.2	0.1	0.0-0.3	0.2	0.2-0.2	0.1	0.1-0.1
OTHER HEALTH CONDITIONS								
Arthritis	47.3	45.5-49.1	64.0	61.7-66.3	36.0	35.8-36.2	46.8	46.5-47.0
Diabetes	46.7	45.1-48.3	40.7	38.8-42.6	40.3	40.1-40.5	34.3	34.1-34.5
Osteoporosis	6.2	5.7-6.9	28.8	27.2-30.6	3.7	3.6-3.7	23.2	22.9-23.4
MENTAL HEALTH CONDITIONS								
Dementia	20.2	19.0-21.5	21.3	19.8-22.9	12.3	12.2-12.5	13.6	13.4-13.8
Depression	35.9	34.4-37.4	42.4	40.5-44.3	16.3	16.1-16.4	25.5	25.3-25.7
Schizophrenia	7.0	6.3-7.7	6.2	5.5-7.0	3.2	3.1-3.3	3.6	3.5-3.6
SUBSTANCE USE DISORDERS								
Alcohol	6.3	5.7-7.0	2.9	2.4-3.5	4.1	4.0-4.2	1.4	1.4-1.4
Drug	3.7	3.2-4.2	4.1	3.5-4.7	1.7	1.7-1.8	1.7	1.6-1.7
Tobacco	11.3	10.5-12.1	9.1	8.2-10.0	8.7	8.5-8.8	5.8	5.7-5.9

TFN, Trans Feminine and Nonbinary; TMN, Trans Masculine and Nonbinary; 95% CI, Confidence Interval. COPD: Chronic obstructive pulmonary disease. This model adjusted for age, race/ethnicity, census region, and months of enrollment. Unclassified individuals were included in the model but not shown for clarity of presentation. For the within- and between-group gender comparisons, 94% were significant at p<0.05 and p<0.01; 91% were significant at p<0.001. There were no significant differences (p>0.05) between TFN and TMN people for: colorectal cancer, lung cancer, congestive heart failure, COPD, dementia, schizophrenia, and drug use disorder. There were no significant differences (p>0.05) between TFN and cisgender females for arthritis. There were no significant differences (p>0.05) between TFN and cisgender males for lung cancer. There were no significant differences (p>0.05) between TMN and cisgender males for: colorectal cancer, lung cancer, cardiac arrhythmia, coronary artery disease, diabetes, HIV/AIDS, and tobacco use disorder. See **Supplemental Table 2** for the estimates and p-values for all comparisons.

When exploring within-group differences in the transgender population, TFN Medicare beneficiaries had a significantly and notably elevated probability of being diagnosed with HIV/AIDS and alcohol use disorder relative to TMN beneficiaries, whereas TMN beneficiaries had a significantly and notably elevated probability of being diagnosed with breast cancer, arthritis, and osteoporosis relative to TFN beneficiaries (all p’s<0.001). With the exception of breast cancer, asthma, arthritis, osteoporosis, and each mental health condition, which were significantly elevated among cisgender females, cisgender males had a higher probability of being diagnosed with all other conditions (p<0.01).

When between-group differences were assessed by those presumed to be assigned a male sex at birth, TFN beneficiaries had a significantly and notably elevated probability of being diagnosed with HIV/AIDS, hepatitis, depression, dementia, schizophrenia, and substance use disorder compared to cisgender male beneficiaries (all p’s<0.001). When exploring between-group differences by those presumed to be assigned a female sex at birth, the widest disparities were observed between TMN and cisgender female beneficiaries for all heart, lung, and kidney diseases, arthritis, hepatitis, depression, dementia, schizophrenia, and substance use disorder diagnoses, with TMN beneficiaries having a significantly elevated probability of all diagnoses (all p’s<0.001) except HIV/AIDS, which was comparable (p=0.64).

When assessing between-group disparities between TFN people and cisgender females, TFN people had a significantly higher probability of having all diagnoses (all p’s<0.001) except arthritis (p<0.55), with the probability of the following diagnoses being particularly elevated: HIV/AIDS, hepatitis, and all substance use disorders. Conversely, TMN people had a significantly higher probability of being diagnosed with breast cancer, arthritis, osteoporosis, depression, and drug use disorder relative to cisgender males (all p’s<0.001). In contrast, cisgender males had a significantly higher probability of alcohol use disorder relative to TMN people (p<0.001).

## Discussion

This study advances the fields of endocrinology and population health research through the application of an algorithm that identifies transgender people in Medicare data and stratifies by inferred gender to explore within- and between-group disparities in health diagnoses among aging transgender and cisgender beneficiaries. We found that overall, aging TFN or TMN Medicare beneficiaries had a significantly higher probability of every health diagnosis studied relative to cisgender male or female beneficiaries, with TFN individuals experiencing some of the highest diagnosis burdens relative to other groups. Future application of these methods will enable the study of rare and hormone and anatomy-related conditions among hard-to-reach aging transgender populations and their cisgender counterparts and can inform interventions to address documented health diagnosis disparities (39).

In understanding the health diagnosis disparities observed here, it is essential to underscore the role of stigma and its stress-related sequelae that transgender individuals differentially experience throughout their lives relative to cisgender individuals (7). Indeed, at the structural level, harmful state, federal, and organizational policies may intentionally or unintentionally restrict access to the essential resources (e.g., healthcare, education, employment, housing, public bathrooms, and other accommodations) that transgender people need to maintain their health (7). Additionally, exposure to interpersonal discrimination, violence, and other sources of enacted stigma at the hands of healthcare providers, family members, employers, educators, sexual partners, and others further restricts access to essential resources and contributes to poor health directly and indirectly through chronic stress (7). As a person ages, chronic activation of the body’s stress response system can compromise health over time *via* a process called allostatic load (40–42). Chronic stress is associated with adverse health outcomes, such as cancer, hypertension, diabetes, mood and substance use disorders, and even death (43, 44), and is therefore theorized to be a primary driver of the health diagnosis disparities observed among aging transgender individuals relative to cisgender individuals in this study (7).

Few large-scale studies have explored disparities within transgender populations, and relative to cisgender males and females for health conditions such as breast cancer that are known to differ by assigned birth sex. In the United States, breast cancer is approximately 70-100 times less common among cisgender males than females (45). Building on prior cancer research with transgender samples (46–50), we found that both TMN people and cisgender females had a significantly elevated probability of a breast cancer diagnosis relative to TFN people and cisgender males. Additionally, although the probability of a breast cancer diagnosis in TFN people was significantly lower than that of cisgender females, it was significantly higher relative to that of cisgender males – a finding that aligns with data from a younger Dutch cohort study comparing the incidence of breast cancer in TFN people on estrogen to cisgender females and males (46). Lifetime stress, a risk factor for cancer (51, 52), together with higher rates of screening among transgender people with breast tissue (53), may have contributed to the elevated probability of being diagnosed with breast cancer among TFN and TMN people relative to their cisgender counterparts. Higher levels of endogenous estradiol in the blood are also associated with a higher risk of breast cancer (54), whereas endogenous androgens are known to inhibit the progression of certain types of breast cancer (46, 55). Decreased testosterone due to the use of antiandrogenic treatment and orchiectomy and denser breast tissue due to the use of exogenous hormones (56) could increase the risk for certain types of breast cancer in TFN people relative to cisgender males (46, 55). Still, the risk of taking exogenous estrogen for gender affirmation is no greater for TFN people than it is for cisgender females taking such therapies for other indications (57–59). Moreover, our study found a significantly lower probability of being diagnosed with breast cancer in TFN people as compared to cisgender females, which may be due to TFN people having less breast tissue and lower lifetime exposure to estrogen than cisgender females.

Notable differences in the probability of osteoporosis and arthritis were also observed among TFN and TMN individuals relative to their cisgender comparators. Specifically, TMN individuals had the highest probability of being diagnosed with osteoporosis and arthritis than all other groups. Further, although the probability of an osteoporosis diagnosis was substantially and significantly lower among TFN people than cisgender females, TFN people had a significantly higher probability of having an osteoporosis diagnosis relative to cisgender males. Our findings align with research showing that osteoporosis and most types of arthritis are more prevalent in older people assigned a female sex at birth than those assigned a male sex at birth (60–62). Obesity, heart disease, and smoking can also increase the risk for rheumatoid arthritis (60, 63), and our study and prior research suggest that TMN people may be at elevated risk of being diagnosed with these conditions (3, 64–66). Relatedly, known risk factors for osteoporosis in the general population include high alcohol consumption, tobacco use, anorexia nervosa, rheumatoid arthritis, chronic kidney disease, and HIV infection (59, 62, 67–69), all of which (except anorexia, which was not studied) were more commonly diagnosed among TFN or TMN people in our sample relative to cisgender males and females. Low physical activity has also been linked to osteoporosis risk in cisgender people (62) and transgender people, even before initiating hormones (59). Research finds that some TFN and TMN individuals have lower levels of physical activity than their cisgender comparators due to various social and physical barriers to exercising, including inadequate changing facilities, revealing and heavily gendered sports clothing, body dissatisfaction, and fears of acceptance (70–72). Additionally, stress is a major risk factor for osteoporosis (62), and other chronic conditions (e.g., tobacco smoking) that increase osteoporosis and arthritis risk (61, 62, 73); and as previously noted, transgender individuals experience greater stigma-related stress throughout their lives relative to cisgender people (7).

Wear and tear on one’s joints and changes in hormone levels in older age are associated with a greater risk of arthritis and osteoporosis, respectively (62, 74, 75). Although there is no evidence to suggest a causal link between exogenous hormone use and arthritis risk in transgender people, in considering osteoporosis risk, the World Professional Association of Transgender Health notes that the use of gender-affirming medical and surgical interventions, such as hormone therapy, androgen blockade, and gonadectomy, have the potential to influence bone health in different ways (59). While testosterone therapy has been associated with no change or even improvements in bone density among TMN people (59, 67, 76), the higher probability of being diagnosed with osteoporosis among TMN people in this sample may be due to natural reductions in estrogen among those who experienced menopause (62, 75). Further, although TFN people have been shown to have improved bone density after initiating estrogen (59, 67, 77, 78), risk factors for osteoporosis include the absence of or underutilization of estrogen after gonadectomy or the use of androgen blockers without or with insufficient estrogen (59, 67, 79). Further, although Gonadotropin-Releasing Hormone Agonists (GnRHa) are very effective in reducing testosterone levels to help TFN individuals achieve their gender-affirmation goals, the use of GnRHA can result in osteoporosis if concurrent doses of estrogen are insufficient (59, 67, 80). In light of prior research, our findings underscore the necessity for clinicians to understand the various physiological, developmental, behavioral, and environmental risk and protective factors for osteoporosis and other health conditions in order to support aging transgender and cisgender individuals in optimizing their health and well-being.

When exploring mental health, in alignment with prior research (5, 12, 13, 30, 59, 81), this study found a significantly higher probability of all mental health diagnoses among both aging TFN and TMN Medicare beneficiaries relative to their cisgender counterparts. Although TFN and TMN people had a similar probability of having a dementia diagnosis, the probability among TFN and TMN people was significantly elevated relative to cisgender males and females. Further, although depression diagnoses were also significantly elevated among TFN and TMN people compared to the cisgender subgroups, both TMN people and cisgender females had a significantly higher probability of depression than TFN people and cisgender males, respectively. Prior research finds that individuals assigned a female sex at birth are twice as likely to be diagnosed with depression than people assigned a male sex at birth (82, 83), which is consistent with our findings. Burgeoning research also finds an elevated probability of Alzheimer’s disease and related dementias (84) and schizophrenia (85) diagnoses among transgender people relative to cisgender individuals. Although the mechanisms underlying these disparities are not well understood, misdiagnoses by untrained or biased providers may contribute to more diagnoses for transgender people, particularly in the case of schizophrenia (59, 60). Additionally, transgender people have historically been required to undergo psychotherapy to be approved for medical gender affirmation treatment (86–88); thus, the higher probability of mental health diagnoses observed here may be due to increased contact with mental health specialists rather than a reflection of the true probability of these conditions in aging transgender populations (85). Nonetheless, extensive research has documented disparities in poor mental health among transgender vs. cisgender individuals (2, 59, 89). Given that the onset of depression, dementia, cognitive decline, and schizophrenia have all been linked to stress in both transgender and cisgender samples (7, 90–96), it is quite probable that the elevated mental health burden observed among aging transgender people in this sample is due to the high levels of stigma-related stress that transgender people experience throughout the life course including in the context of receiving medical care (7, 26, 97).

Some of the most striking within- and between- gender group diagnostic disparities were observed with regard to infectious diseases. The probability of HIV/AIDS among TFN people was approximately 9-fold that of TMN people and cisgender females and 4.5-fold that of cisgender males. The high probability of HIV/AIDS among TFN people is well documented in the literature (4, 12, 13, 98) and has been attributed to multilevel factors. For example, TFN people may acquire HIV at higher rates due to networks-level factors such as the high prevalence of HIV within TFN people’s limited pool of potential sexual partners or anatomical considerations that predispose TFN people with a penis to engage in sexual acts that carry greater HIV risk (i.e., receptive anal sex) (99, 100). A confluence of stigma-related, structural, and interpersonal factors also restrict access to essential human needs (i.e., employment, shelter, food, love, gender affirmation) for TFN people (7) and can lead to engagement in HIV risk behavior such as transactional sex for financial survival (99, 100), receptive anal sex (i.e., “bottoming”) as a means to be affirmed in one’s gender when access to other sources of affirmation are limited (100–102), and condomless sex to please one’s sexual partner in the context of relationship stigma and partner scarcity (100, 103, 104). Similarly, the probability of hepatitis was significantly higher among TFN people than all other groups, and the probability of a hepatitis diagnosis among TMN people was roughly twice that of cisgender males and females. In addition to the aforementioned sexual pathways, the significantly higher probability of a hepatitis diagnosis among transgender people relative to cisgender individuals may also be driven by the sharing of syringes for injecting drugs, hormones, and silicone - behaviors that may be more prevalent among transgender people due to stigma-related barriers to healthcare (7, 99–101, 105–107).

Consistent with prior research with Medicare- and commercially-insured individuals (3, 4, 12, 13, 30), aging transgender Medicare beneficiaries in this sample had a significantly higher probability of most substance use disorder diagnoses than their cisgender comparators. Research has consistently found elevated levels of substance use and diagnosed substance use disorders among transgender samples relative to cisgender samples (3, 5, 12, 13, 30, 108), due in part to the need to cope with the psychological toll of discrimination, violence, and other forms of stigma (1, 7, 34, 109). In prior studies of younger, commercially-insured transgender individuals (3, 4, 12, 13), drug, alcohol, and tobacco use disorder diagnoses were particularly elevated among TFN people than TMN people. In contrast, the probability of having a drug use disorder diagnosis was fairly comparable between TFN and TMN people in the current study, though the probability of alcohol and tobacco use disorder diagnoses was significantly elevated among TFN people relative to TMN people in the sample. A similar trend was observed for cisgender individuals, such that cisgender males had a significantly higher probability of alcohol and tobacco use disorder diagnoses than cisgender females. The substance use disorder findings suggest that although stigma likely drives the higher prevalence of substance use disorders observed among transgender people (7), individuals assigned a male sex at birth may be more susceptible to some substance use disorders than those assigned a female sex at birth (110, 111). It is also possible that developmental factors may lead individuals assigned a male sex at birth to cope with stress using certain substances more frequently than those assigned a female sex at birth (110, 111). Although the receipt of a diagnosis is an important step in linkage to treatment, research finds that transgender people face difficulty finding affirming substance use treatment services (112–114). Thus, efforts are needed to effectively prevent and treat substance use disorders among transgender people by reducing societal stigma (7), helping transgender people to cope with stigma through health-promoting means (115, 116), and improving access to gender-affirming substance use treatment services (117).

### Clinical and research implications

Although TFN or TMN aging adults in our sample had a significantly higher probability of receiving specific health diagnoses relative to one or both cisgender subgroups, current, evidenced-based clinical care guidelines (59) show that the use of exogenous hormones carries similar risks for various health outcomes in both transgender and cisgender populations (e.g., risk of breast cancer in TFN people taking estrogen is no greater than for cisgender women taking estrogen for other indications) (57–59). Thus, research such as ours, which shows disparities in health diagnoses rather than the true prevalence of disease in transgender and cisgender people, should not be used to create differential access to medically-necessary treatment for transgender people (59, 87, 88).

Significant advancements in endocrinology and transgender medicine in recent years have helped to ensure the safe and effective delivery of medically-necessary gender-affirming hormones and surgical treatments to transgender people and have led to improvements in the psychological well-being and quality of life for transgender individuals accessing such care (59, 118). Given that this study focuses on aging transgender individuals, it is possible that some of the health diagnosis disparities observed here are due to their past utilization of older treatment regimens prior to the advent of quality care guidelines, as well as a confluence of stigma-related social, behavioral, and environmental risk factors that shaped access to and use of quality gender-affirming medical care (7). Our findings, together with prior research, underscore the need for high-quality, prospective research to identify the multitude of risk factors that may contribute to the health diagnosis disparities observed here. Such research should include ongoing, rigorous examinations of the risk and protective effects of gender-affirming hormone therapy and surgery and evaluations into whether transgender individuals are able to access guideline-concordant care. Such data can help to [1] improve healthcare providers’ ability to provide quality care to transgender patients; [2] ensure that transgender individuals receive the requisite information to provide fully informed consent when accessing medically-necessary and psychologically-beneficial medications and procedures to affirm their gender (59, 118, 119); and [3] inform clinical and policy interventions aimed at improving the health and well-being of transgender people.

### Limitations

The study has limitations. First, since transgender beneficiaries were included in our study based on their observed care, we are unable to validate whether individuals we identified as transgender were truly so or whether individuals we characterized as cisgender based on their absence of qualifying care were actually cisgender. Second, since our administrative data did not capture gender identity, we were forced to infer the gender identity of our sample and combine individuals who likely hold nonbinary and binary gender identities in the same category based on their shared use of certain gender-affirming hormones, procedures, or anatomy-specific care. Nonbinary transgender people have been shown to have differential healthcare utilization and risk of various health outcomes than binary transgender people (32, 120), and so the necessary combining of these groups in the present study may have obscured key differences in the probability of being diagnosed with one or more health conditions. Third, transgender and cisgender beneficiaries were included in our study based on their observed care, whereas individuals who did not access relevant care through fee-for-service Medicare at any point during the study period were not included; thus, our sample is unlikely to represent all aging transgender and cisgender Medicare beneficiaries. Fourth, our estimated burden of health diagnoses was based on a methodology that relies on healthcare utilization, and the longer individuals were enrolled in Medicare, the more time they had to engage in healthcare and receive a diagnosis. Since transgender individuals, on average, had more months of enrollment in fee-for-service Medicare than cisgender individuals, we adjusted for months of enrollment. However, undiagnosed or underdiagnosed conditions within any of the gender groups would result in an undercount of the true burden of disease. Further, as noted earlier, transgender individuals forced reliance on the healthcare system to receive gender-affirming care may have led to higher healthcare utilization and more opportunities to receive a health diagnosis relative to cisgender people. Fifth, our analyses may be prone to bias due to unmeasured confounding. Specifically, the effect of social risk factors such as education, housing stability, and income could not be assessed using our data. Still, our findings align with and extend other claims-based, survey, and clinical studies and act as a signal for future research and intervention efforts.

## Conclusion

We adapted prior algorithms (3, 13, 33) to identify a large sample of aging transgender Medicare beneficiaries and examine within- and between-group differences in health diagnoses among transgender people and their cisgender counterparts. Extending prior research (3, 5, 12, 13, 30, 31, 33), we observed an elevated burden of health condition diagnoses among TFN or TMN people overall, with the greatest burden observed among TFN people relative to other groups. Our novel methods to identify a transgender sample using Medicare claims data and infer gender may be helpful for future researchers seeking to study the diagnosis of rare conditions, as well as identify transgender subgroups in need of preventive and treatment interventions aimed at reducing morbidity (26, 29, 30, 32) and mortality (42, 121) among aging transgender people in the U.S.

## Data availability statement

The original contributions presented in the study are included in the article/**Supplementary Material**. Further inquiries can be directed to the corresponding author.

## Ethics statement

This study was approved by the Brown University Institutional Review Board. Written informed consent from the participants was not required to participate in this study in accordance with national legislation and institutional requirements.

## Author contributions

JH and TS conceived the project and wrote the grants that funded access to the data and supported the analyst's time. JH, TS, AA, JE, LH, KY, and JD were involved in developing the methods to identify the transgender sample and stratify the sample by inferred gender. All authors were involved in refining the algorithm. JH wrote and edited the manuscript and designed and edited the tables and supplementary figures. HV conducted the analyses and populated the tables. KY and LH reviewed the statistical code. GB contributed to the writing of the introduction and formatting of the paper. KY created **Figure 1**. All authors contributed to the article and approved the submitted version.

## Footnotes

1 Yee, Lind, & Downing (2021) used inferred sex assigned birth sex instead of inferred gender identity when stratifying the transgender sample.

2 Reproductive-anatomy-related care was previously referred to as “sex-specific care”. Since people assigned the same sex at birth may have different anatomies, we revised the term to be more specific to anatomy.

3 It is not possible to determine the true gender identity or assigned birth sex of individuals identified in claims data. Drawing on Jasuja and colleagues’ formative research and consultations with transgender clinicians, researchers, and community advisory board members and extending our prior work, we categorized the transgender sample based on inferred gender as opposed to inferred sex assigned at birth. We considered inferred gender population. There was also the concern that referring to transgender subgroups based on their assigned birth sex would effectively sanction the practice of referring to transgender people by their assigned birth sex as opposed to their self-reported gender identity in clinical and other encounters, which is not affirming or appropriate.

4 TFN people represent people who take gender-affirming hormones or who have received gender-affirming procedures to achieve a nonbinary-to-feminine gender expression and/or who have had other types of diagnoses, or reproductive anatomy-specific procedures common to someone assigned a male birth sex (e.g., prostate-related care). TMN people represent people taking gender-affirming hormones or who have received gender-affirming procedures to achieve a nonbinary-to-masculine gender expression and/or who have had other types of diagnoses, or reproductive anatomy-related procedures common to someone assigned a female birth sex (e.g., hysterectomy).
