# Supplementary material for: Disparities in health condition diagnoses among aging transgender and cisgender medicare beneficiaries, 2008-2017
Source: Front Endocrinol (Lausanne). 2023 Mar 13;14:1102348. doi: 10.3389/fendo.2023.1102348 (PMC10040837; doi:10.3389/fendo.2023.1102348)
Supplement: Supplementary file 1 [file Table_1.pdf]

Supplemental Table 1. Unadjusted prevalence of health condition diagnoses stratified by inferred gender in a national age-entitled Medicare population, 2008-2017

	TRANSGENDER						CISGENDER			
	TFN N=4,198		TMN N=2,762		Unclassified N=3,015		Male N=1,294,690		Female N=1,666,946	
	N	%	N	%	N	%	N	%	N	%
**CANCER**										
Breast	26	0.6%	367	13.3%	122	4.0%	2,029	0.2%	141,699	8.5%
Colorectal	204	4.9%	132	4.8%	101	3.3%	42,742	3.3%	48,351	2.9%
Endometrial	---	---	85	3.1%	---	---	---	---	25,281	1.5%
Lung	151	3.6%	114	4.1%	94	3.1%	41,659	3.2%	40,460	2.4%
Prostate	698	16.6%	---	---	---	---	159,196	12.3%	---	---
**HEART, LUNG, & KIDNEY CONDITIONS**										
Asthma	706	16.8%	656	23.8%	516	17.1%	109,325	8.4%	211,096	12.7%
Cardiac Arrhythmia	1,046	24.9%	675	24.4%	612	20.3%	227,603	17.6%	246,487	14.8%
Chronic Kidney Disease	2,101	50.0%	1,320	47.8%	1,252	41.5%	406,431	31.4%	454,226	27.2%
Congestive Heart Failure	1,619	38.6%	1,167	42.3%	1,135	37.6%	353,371	27.3%	452,657	27.2%
COPD	1,587	37.8%	1,111	40.2%	1,076	35.7%	321,038	24.8%	392,914	23.6%
Coronary Artery Disease	2,505	59.7%	1,589	57.5%	1,492	49.5%	587,029	45.3%	637,958	38.3%
Hyperlipemia	3,430	81.7%	2,274	82.3%	1,996	66.2%	788,727	60.9%	1,025,980	61.5%
Hypertension	3,495	83.3%	2,346	84.9%	2,252	74.7%	859,723	66.4%	1,117,285	67.0%
Stroke	883	21.0%	692	25.1%	591	19.6%	189,082	14.6%	262,262	15.7%
**INFECTIOUS DISEASES**										
Hepatitis	149	3.5%	73	2.6%	101	3.3%	16,028	1.2%	14,707	0.9%
HIV/AIDS	42	1.0%	< 11	---	38	1.3%	2,396	0.2%	1,184	0.1%
**OTHER HEALTH CONDITIONS**										
Arthritis	2,484	59.2%	2,088	75.6%	1,537	51.0%	496,412	38.3%	831,289	49.9%
Diabetes	1,884	44.9%	1,182	42.8%	1,108	36.7%	427,602	33.0%	502,215	30.1%
Osteoporosis	475	11.3%	1,172	42.4%	774	25.7%	66,004	5.1%	473,972	28.4%
**MENTAL HEALTH CONDITIONS**										
Dementia	1,054	25.1%	839	30.4%	897	29.8%	210,079	16.2%	356,774	21.4%
Depression	1,946	46.4%	1,550	56.1%	1,395	46.3%	260,171	20.1%	534,063	32.0%
Schizophrenia	418	10.0%	293	10.6%	344	11.4%	59,746	4.6%	105,263	6.3%
**SUBSTANCE USE DISORDERS**										
Alcohol	365	8.7%	121	4.4%	165	5.5%	59,720	4.6%	27,265	1.6%
Drug	250	6.0%	201	7.3%	195	6.5%	28,843	2.2%	39,069	2.3%
Tobacco	729	17.4%	416	15.1%	485	16.1%	144,113	11.1%	129,175	7.7%

Note. TFN = Trans Feminine and Nonbinary; TMN = Trans Masculine and Nonbinary. Cells with an N of 11 or less are suppressed.
