# Supplementary material for: Disparities in health condition diagnoses among aging transgender and cisgender medicare beneficiaries, 2008-2017
Source: Front Endocrinol (Lausanne). 2023 Mar 13;14:1102348. doi: 10.3389/fendo.2023.1102348 (PMC10040837; doi:10.3389/fendo.2023.1102348)
Supplement: Supplementary file 2 [file Table_2.pdf]

Supplemental Table 2. Mean differences in the predicted probabilities of health condition diagnoses by inferred gender in a national age-entitled Medicare population, 2008-2017.

Condition	Group 1	Group 2	Estimate	95% CI		P-Value
**CANCER**						
Breast	TFN	TMN	-3.13	-3.53	-2.73	**<.0001**
	TFN	Cisgender Male	1.13	0.74	1.52	**<.0001**
	TFN	Cisgender Female	-2.92	-3.31	-2.53	**<.0001**
	TMN	Cisgender Male	4.25	4.13	4.37	**<.0001**
	TMN	Cisgender Female	0.21	0.10	0.32	**0.0003**
	Cisgender Male	Cisgender Female	-4.05	-4.09	-4.00	**<.0001**
Colorectal	TFN	TMN	0.15	-0.07	0.38	0.18
	TFN	Cisgender Male	0.28	0.13	0.42	**0.0001**
	TFN	Cisgender Female	0.55	0.41	0.69	**<.0001**
	TMN	Cisgender Male	0.12	-0.05	0.30	0.17
	TMN	Cisgender Female	0.40	0.22	0.57	**<.0001**
	Cisgender Male	Cisgender Female	0.27	0.26	0.29	**<.0001**
Endometrial	TMN	Cisgender Female	0.45	0.24	0.67	**<.0001**
Lung	TFN	TMN	-0.08	-0.33	0.17	0.51
	TFN	Cisgender Male	0.09	-0.07	0.25	0.27
	TFN	Cisgender Female	0.42	0.26	0.58	**<.0001**
	TMN	Cisgender Male	0.18	-0.01	0.36	0.07
	TMN	Cisgender Female	0.51	0.32	0.69	**<.0001**
	Cisgender Male	Cisgender Female	0.33	0.32	0.34	**<.0001**
Prostate	TFN	Cisgender Male	0.11	0.03	0.20	**0.01**
**HEART, LUNG, & KIDNEY CONDITIONS**						
Asthma	TFN	TMN	-0.32	-0.45	-0.20	**<.0001**
	TFN	Cisgender Male	0.52	0.44	0.60	**<.0001**
	TFN	Cisgender Female	0.14	0.06	0.23	**0.001**
	TMN	Cisgender Male	0.84	0.75	0.93	**<.0001**
	TMN	Cisgender Female	0.47	0.38	0.56	**<.0001**
	Cisgender Male	Cisgender Female	-0.38	-0.38	-0.37	**<.0001**
Cardiac Arrhythmia	TFN	TMN	0.22	0.10	0.34	**0.0003**
	TFN	Cisgender Male	0.25	0.17	0.32	**<.0001**
	TFN	Cisgender Female	0.67	0.59	0.74	**<.0001**
	TMN	Cisgender Male	0.03	-0.06	0.12	0.52
	TMN	Cisgender Female	0.45	0.36	0.54	**<.0001**
	Cisgender Male	Cisgender Female	0.42	0.41	0.43	**<.0001**
Chronic Kidney Disease	TFN	TMN	0.31	0.21	0.42	**<.0001**
	TFN	Cisgender Male	0.57	0.50	0.63	**<.0001**
	TFN	Cisgender Female	0.96	0.90	1.03	**<.0001**
	TMN	Cisgender Male	0.25	0.17	0.33	**<.0001**
	TMN	Cisgender Female	0.65	0.57	0.73	**<.0001**
	Cisgender Male	Cisgender Female	0.40	0.39	0.41	**<.0001**
Congestive Heart Failure	TFN	TMN	0.04	-0.07	0.15	0.43
	TFN	Cisgender Male	0.44	0.37	0.51	**<.0001**
	TFN	Cisgender Female	0.69	0.62	0.76	**<.0001**

	TMN	Cisgender Male	0.40	0.31	0.48	**<.0001**
	TMN	Cisgender Female	0.65	0.56	0.73	**<.0001**
	Cisgender Male	Cisgender Female	0.25	0.24	0.26	**<.0001**
COPD	TFN	TMN	0.06	-0.05	0.16	0.28
	TFN	Cisgender Male	0.44	0.38	0.51	**<.0001**
	TFN	Cisgender Female	0.66	0.60	0.73	**<.0001**
	TMN	Cisgender Male	0.38	0.30	0.47	**<.0001**
	TMN	Cisgender Female	0.61	0.53	0.69	**<.0001**
	Cisgender Male	Cisgender Female	0.22	0.22	0.23	**<.0001**
Coronary Artery Disease	TFN	TMN	0.35	0.24	0.46	**<.0001**
	TFN	Cisgender Male	0.33	0.26	0.40	**<.0001**
	TFN	Cisgender Female	0.90	0.83	0.97	**<.0001**
	TMN	Cisgender Male	-0.02	-0.10	0.06	0.64
	TMN	Cisgender Female	0.55	0.46	0.63	**<.0001**
	Cisgender Male	Cisgender Female	0.57	0.56	0.57	**<.0001**
Hyperlipemia	TFN	TMN	0.29	0.14	0.43	**<.0001**
	TFN	Cisgender Male	0.48	0.39	0.57	**<.0001**
	TFN	Cisgender Female	0.57	0.49	0.66	**<.0001**
	TMN	Cisgender Male	0.19	0.08	0.30	**0.0007**
	TMN	Cisgender Female	0.29	0.18	0.40	**<.0001**
	Cisgender Male	Cisgender Female	0.10	0.09	0.10	**<.0001**
Hypertension	TFN	TMN	0.19	0.04	0.34	**0.01**
	TFN	Cisgender Male	0.48	0.39	0.57	**<.0001**
	TFN	Cisgender Female	0.61	0.52	0.70	**<.0001**
	TMN	Cisgender Male	0.29	0.17	0.41	**<.0001**
	TMN	Cisgender Female	0.42	0.30	0.54	**<.0001**
	Cisgender Male	Cisgender Female	0.13	0.13	0.14	**<.0001**
Stroke	TFN	TMN	-0.05	-0.18	0.07	0.37
	TFN	Cisgender Male	0.29	0.21	0.37	**<.0001**
	TFN	Cisgender Female	0.40	0.32	0.48	**<.0001**
	TMN	Cisgender Male	0.34	0.25	0.44	**<.0001**
	TMN	Cisgender Female	0.45	0.36	0.54	**<.0001**
	Cisgender Male	Cisgender Female	0.11	0.10	0.11	**<.0001**
**INFECTIOUS DISEASES**						
Hepatitis	TFN	TMN	0.39	0.10	0.68	**0.008**
	TFN	Cisgender Male	0.90	0.74	1.07	**<.0001**
	TFN	Cisgender Female	1.30	1.14	1.47	**<.0001**
	TMN	Cisgender Male	0.51	0.28	0.75	**<.0001**
	TMN	Cisgender Female	0.91	0.67	1.15	**<.0001**
	Cisgender Male	Cisgender Female	0.40	0.38	0.42	**<.0001**
HIV/AIDS	TFN	TMN	2.32	1.15	3.50	**0.0001**
	TFN	Cisgender Male	1.55	1.24	1.87	**<.0001**
	TFN	Cisgender Female	2.59	2.27	2.90	**<.0001**
	TMN	Cisgender Male	-0.77	-1.90	0.37	0.18
	TMN	Cisgender Female	0.27	-0.87	1.40	0.64
	Cisgender Male	Cisgender Female	1.04	0.97	1.11	**<.0001**

**OTHER HEALTH CONDITIONS**						
Arthritis	TFN	TMN	-0.69	-0.81	-0.57	**<.0001**
	TFN	Cisgender Male	0.47	0.40	0.54	**<.0001**
	TFN	Cisgender Female	0.02	-0.05	0.09	0.55
	TMN	Cisgender Male	1.15	1.05	1.25	**<.0001**
	TMN	Cisgender Female	0.71	0.61	0.81	**<.0001**
	Cisgender Male	Cisgender Female	-0.44	-0.45	-0.44	**<.0001**
Diabetes	TFN	TMN	0.24	0.14	0.34	**<.0001**
	TFN	Cisgender Male	0.26	0.20	0.32	**<.0001**
	TFN	Cisgender Female	0.52	0.45	0.58	**<.0001**
	TMN	Cisgender Male	0.02	-0.06	0.10	0.65
	TMN	Cisgender Female	0.27	0.19	0.35	**<.0001**
	Cisgender Male	Cisgender Female	0.25	0.25	0.26	**<.0001**
Osteoporosis	TFN	TMN	-1.81	-1.94	-1.68	**<.0001**
	TFN	Cisgender Male	0.56	0.46	0.66	**<.0001**
	TFN	Cisgender Female	-1.51	-1.61	-1.41	**<.0001**
	TMN	Cisgender Male	2.37	2.28	2.45	**<.0001**
	TMN	Cisgender Female	0.30	0.21	0.38	**<.0001**
	Cisgender Male	Cisgender Female	-2.07	-2.08	-2.06	**<.0001**
**MENTAL HEALTH CONDITIONS**						
Dementia	TFN	TMN	-0.07	-0.19	0.06	0.29
	TFN	Cisgender Male	0.59	0.51	0.67	**<.0001**
	TFN	Cisgender Female	0.48	0.40	0.56	**<.0001**
	TMN	Cisgender Male	0.66	0.56	0.75	**<.0001**
	TMN	Cisgender Female	0.54	0.45	0.64	**<.0001**
	Cisgender Male	Cisgender Female	-0.11	-0.12	-0.11	**<.0001**
Depression	TFN	TMN	-0.27	-0.37	-0.17	**<.0001**
	TFN	Cisgender Male	1.06	0.99	1.12	**<.0001**
	TFN	Cisgender Female	0.49	0.43	0.56	**<.0001**
	TMN	Cisgender Male	1.33	1.25	1.41	**<.0001**
	TMN	Cisgender Female	0.76	0.68	0.84	**<.0001**
	Cisgender Male	Cisgender Female	-0.57	-0.57	-0.56	**<.0001**
Schizophrenia	TFN	TMN	0.13	-0.03	0.29	0.12
	TFN	Cisgender Male	0.82	0.72	0.93	**<.0001**
	TFN	Cisgender Female	0.71	0.60	0.81	**<.0001**
	TMN	Cisgender Male	0.69	0.57	0.82	**<.0001**
	TMN	Cisgender Female	0.58	0.45	0.71	**<.0001**
	Cisgender Male	Cisgender Female	-0.11	-0.13	-0.10	**<.0001**
**SUBSTANCE USE DISORDERS**						
Alcohol	TFN	TMN	0.80	0.59	1.01	**<.0001**
	TFN	Cisgender Male	0.45	0.35	0.56	**<.0001**
	TFN	Cisgender Female	1.56	1.45	1.67	**<.0001**
	TMN	Cisgender Male	-0.34	-0.53	-0.16	**0.0002**
	TMN	Cisgender Female	0.76	0.58	0.94	**<.0001**
	Cisgender Male	Cisgender Female	1.10	1.09	1.12	**<.0001**
Drug	TFN	TMN	-0.11	-0.30	0.08	0.26

	TFN	Cisgender Male	0.78	0.65	0.91	**<.0001**
	TFN	Cisgender Female	0.80	0.67	0.93	**<.0001**
	TMN	Cisgender Male	0.89	0.75	1.04	**<.0001**
	TMN	Cisgender Female	0.91	0.77	1.06	**<.0001**
	Cisgender Male	Cisgender Female	0.02	0.01	0.04	**0.007**
Tobacco	TFN	TMN	0.24	0.11	0.37	**0.0004**
	TFN	Cisgender Male	0.29	0.21	0.37	**<.0001**
	TFN	Cisgender Female	0.72	0.64	0.80	**<.0001**
	TMN	Cisgender Male	0.05	-0.05	0.16	0.34
	TMN	Cisgender Female	0.48	0.37	0.58	**<.0001**
	Cisgender Male	Cisgender Female	0.42	0.42	0.43	**<.0001**

Notes. TFN = Trans Feminine and Nonbinary; TMN = Trans Masculine and Nonbinary; CI=95% Confidence Intervals. Bolded variables significant at $p<0.05$.
