# Supplementary figures and images for: Disparities in health condition diagnoses among aging transgender and cisgender medicare beneficiaries, 2008-2017
Source: Front Endocrinol (Lausanne). 2023 Mar 13;14:1102348. doi: 10.3389/fendo.2023.1102348 (PMC10040837; doi:10.3389/fendo.2023.1102348)

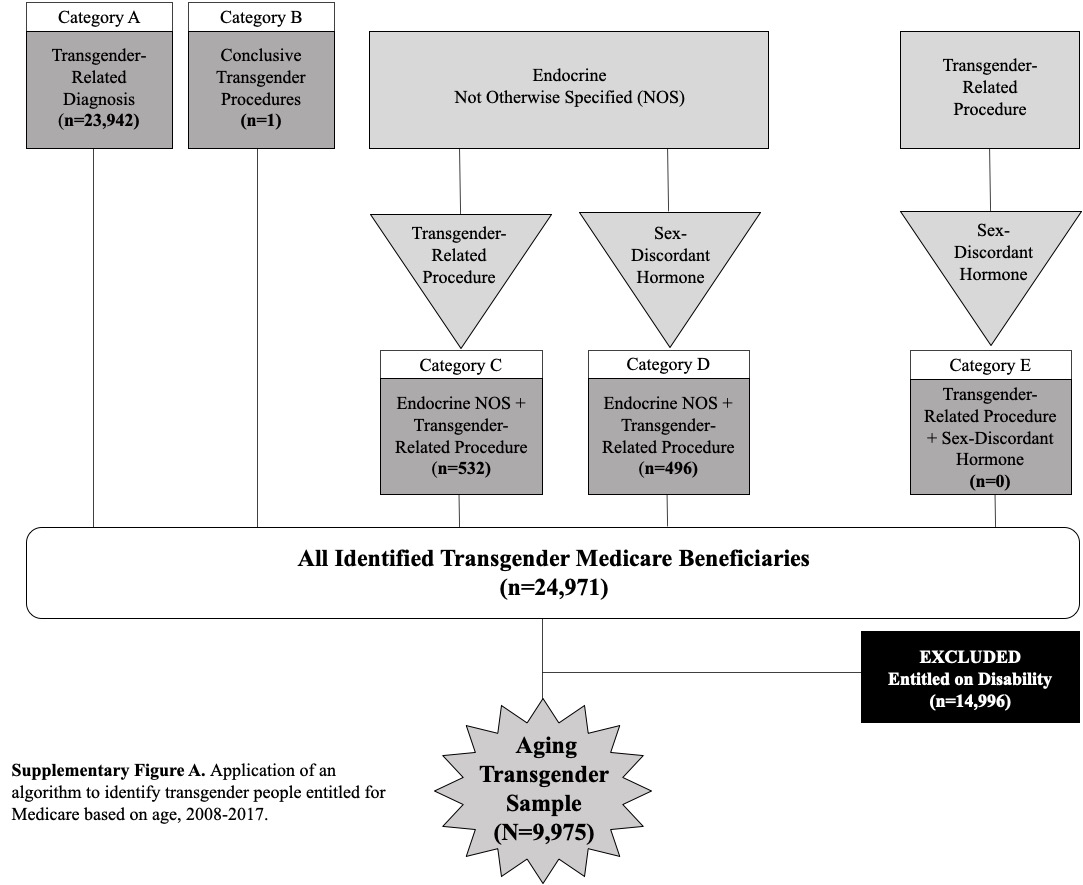

Supplement: Supplementary file 3 [file Image_1.jpeg]

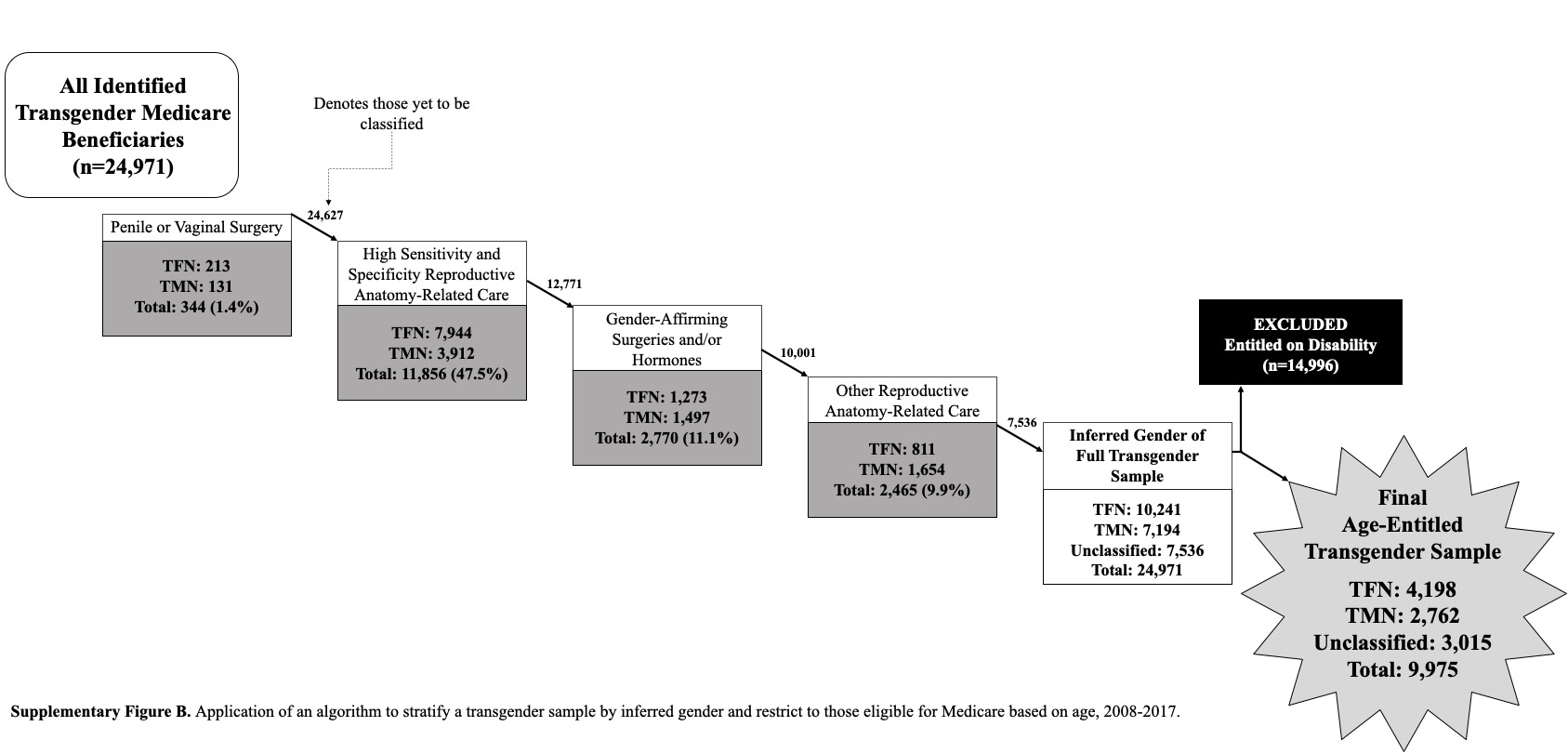

Supplement: Supplementary file 4 [file Image_2.jpeg]
